# Association between internal migration and epidemic dynamics: an analysis of cause-specific mortality in Kenya and South Africa using health and demographic surveillance data

**DOI:** 10.1186/s12889-018-5851-5

**Published:** 2018-07-27

**Authors:** Carren Ginsburg, Philippe Bocquier, Donatien Béguy, Sulaimon Afolabi, Kathleen Kahn, David Obor, Frank Tanser, Andrew Tomita, Marylene Wamukoya, Mark A. Collinson

**Affiliations:** 1 Medical Research Council/Wits Rural Public Health and Health Transitions Research Unit (Agincourt), School of Public Health, Faculty of Health Sciences, University of the Witwatersrand, 27 St Andrews Road, Parktown, Johannesburg, 2193 South Africa; 2 INDEPTH Network, Accra, Ghana; 3 Centre de Recherches en Démographie, Université Catholique de Louvain, Louvain-la-Neuve, Belgium; 4 African Population and Health Research Centre, Nairobi, Kenya; 5 Umeå Centre for Global Health Research, Umeå University, Umeå, Sweden; 6 KEMRI & CDC - Centre for Global Health Research, Kisumu, Kenya; 7 Africa Health Research Institute, University of KwaZulu-Natal, Durban, South Africa; 8 Centre for the AIDS Programme of Research in South Africa (CAPRISA), Durban, South Africa; 9 School of Nursing and Public Health, University of KwaZulu-Natal, Durban, South Africa; 10 Nelson R Mandela School of Medicine, University of KwaZulu-Natal, Durban, South Africa; 11 Department of Science and Technology/ Medical Research Council, South African Population Research Infrastructure Network, Johannesburg, South Africa

**Keywords:** Internal migration, AIDS/TB, NCDs, Mortality, South Africa, Kenya, Health and demographic surveillance system

## Abstract

**Background:**

Many low- and middle-income countries are facing a double burden of disease with persisting high levels of infectious disease, and an increasing prevalence of non-communicable disease (NCD). Within these settings, complex processes and transitions concerning health and population are underway, altering population dynamics and patterns of disease. Understanding the mechanisms through which changing socioeconomic and environmental contexts may influence health is central to developing appropriate public health policy. Migration, which involves a change in environment and health exposure, is one such mechanism.

**Methods:**

This study uses Competing Risk Models to examine the relationship between internal migration and premature mortality from AIDS/TB and NCDs. The analysis employs 9 to 14 years of longitudinal data from four Health and Demographic Surveillance Systems (HDSS) of the INDEPTH Network located in Kenya and South Africa (populations ranging from 71 to 223 thousand). The study tests whether the mortality of migrants converges to that of non-migrants over the period of observation, controlling for age, sex and education level.

**Results:**

In all four HDSS, AIDS/TB has a strong influence on overall deaths. However, in all sites the probability of premature death (45q15) due to AIDS/TB is declining in recent periods, having exceeded 0.39 in the South African sites and 0.18 in the Kenyan sites in earlier years. In general, the migration effect presents similar patterns in relation to both AIDS/TB and NCD mortality, and shows a migrant mortality disadvantage with no convergence between migrants and non-migrants over the period of observation. Return migrants to the Agincourt HDSS (South Africa) are on average four times more likely to die of AIDS/TB or NCDs than are non-migrants. In the Africa Health Research Institute (South Africa) female return migrants have approximately twice the risk of dying from AIDS/TB from the year 2004 onwards, while there is a divergence to higher AIDS/TB mortality risk amongst female migrants to the Nairobi HDSS from 2010.

**Conclusion:**

Results suggest that structural socioeconomic issues, rather than epidemic dynamics are likely to be associated with differences in mortality risk by migrant status. Interventions aimed at improving recent migrant’s access to treatment may mitigate risk.

**Electronic supplementary material:**

The online version of this article (10.1186/s12889-018-5851-5) contains supplementary material, which is available to authorized users.

## Background

The classic framework of epidemiological transition describes the shift from infectious diseases to non-communicable diseases (NCDs) as population income increases and mortality levels decline [1–4]. However, many low- and middle-income countries are facing a double burden of disease with persisting high levels of infectious disease, coupled with an increasing prevalence of NCD [5, 6]. The 2010 Global Burden of Disease study reported that infectious diseases together with maternal, neonatal and nutritional disorders were amongst the leading causes of premature death in sub-Saharan Africa, accounting for 76% of mortality in under 80 year-olds [2]. Cardiovascular disease burden nevertheless increased in sub-Saharan Africa, with death from cardiovascular disease rising by 81% between 1990 and 2013 [7, 8]. Fast-changing health and connected demographic transitions are underway in these settings altering population dynamics and patterns of health and disease [4]. Empirical evidence concerning the dynamics of epidemics and related population trends has highlighted marked regional and national variability [2, 7], however, these processes have been insufficiently documented due to a scarcity of appropriate data.

It is well established that health transitions and epidemic dynamics are influenced by socioenvironmental and economic conditions [9]. Understanding the shifting cause-pattern of disease and mortality [1], as well as the mechanisms through which changing socioeconomic and environmental contexts may influence health is therefore crucial. Migration, which involves a change in environment and health exposure, is one such mechanism. Migration has been recognised as a key risk factor for infectious as well as chronic disease, and operates through complex pathways.

The migration and health relationship is multi-faceted and bi-directional. Health status can drive a decision to migrate, but such relocation may in turn have an influence on health. A number of hypotheses concerning the relationship between migration and health have been proposed in the literature, each relating to a stage of the migration process [10]. The most utilised hypothesis of selection holds that migrants are often amongst the healthier in the population when they undertake a migration (the “healthy migrant hypothesis”) [10]. A paradox is present since migrants who return to their places of origin have been observed to be amongst the less healthy, resulting in a “salmon bias” effect, first described with respect to the return migration of Hispanic migrants from the United States [11]. Similarly studies of internal migrants in sub-Saharan Africa have described the phenomenon of migrants “returning home to die” as their health in destinations deteriorates [12–14].

While migrants may be relatively healthier when they undertake a move, health status may be affected following migration as a result of disruption and stress, behavioural change, unstable employment and possible exposure to disease in a new environment [15–18]. Migrants may encounter barriers in accessing health care, or be exposed to changing lifestyle factors that negatively affect their health and well-being at destinations [16, 19]. These potential risks may be offset by improved employment and education opportunities, or superior health services and infrastructure at migrant destinations - conditions often associated with urban environments [18]. Nevertheless, the balance between potential negative and positive effects of relocation may vary considerably across settings [20].

Duration of residence in a new location has been found to reduce these effects as migrants adapt to their new environment. The hypothesis of adaptation suggests that over time, migrants’ health and behaviours may assimilate to non-migrants in a destination area, resulting in no discernible difference in health status between migrants and non-migrants [10, 16, 21].

Migration, in particular circular migration, has further been linked to the spread of health conditions or behaviours between origin and destination areas. Studies have revealed how infectious diseases such as HIV may be diffused through a population via migration [22, 23]. This has been attributed to the transmission of infection through physical contact and increased sexual risk behaviours associated with mobility [22, 24]. Non-communicable disease risk has been associated with the process of urbanisation where movement to urban areas may expose migrants to negative lifestyle factors such as unhealthy diets or lower levels of physical activity [19, 25, 26], and these lifestyle behavioural changes may potentially spread to other areas via return migration. The role of migration in the propagation of disease will depend on the stage of the epidemic, the prevalence of the disease in an area, and patterns of movements linking origins and destinations [22, 24]. Evidence on the association between migration and epidemic dynamics, and the pathways linking movement to disease is much needed to improve current understanding of interactions between disease and the social and environmental context [27]. Indeed, intergovernmental organisations and researchers have highlighted the importance of focused regional studies, and the triangulation of varied data sources on burden of disease and surrounding processes such as migration in informing the development of appropriate public health policy and interventions [7, 28].

This paper follows from a previous study of the relationship between internal migration and all-cause mortality in nine Health and Demographic Surveillance System (HDSS) sites in sub-Saharan Africa [21]. The study confirmed that premature adult mortality differed strongly by migration status. It also confirmed the adaptation hypothesis by showing that in the ten-year period after migration, no health differences between migrants and non-migrants were observed in these HDSS populations.

This paper explores the relationship between internal migration and mortality by cause of death through an analysis of four HDSS sites situated in Kenya and South Africa. These are two sub-Saharan African countries that present with high rates of premature adult mortality associated with two primary causes: AIDS/TB and NCDs. The study asks how internal migration status relates to patterns of AIDS/TB and NCD mortality in the context of the double burden of disease in these countries. The paper has two objectives. The first objective is to define for each geographical area, the patterns of AIDS/TB and NCD mortality for a period of nine to fourteen years. The second objective is to test whether the mortality of migrants converges to that of non-migrants as the epidemic evolves over the period of observation.

We hypothesise that health consequences of migration are associated with the extent of diffusion of an epidemic in a particular local population. At the initial phase of the epidemic, we would expect the health of migrants to diverge from that of the host population. As the epidemic is diffused, we anticipate that the health of migrants would converge with that of non-migrants as both origin and destination areas have been affected. This pattern of divergence and convergence would represent an association between migrant status and the dynamics of the epidemic. The alternate hypothesis is that the health consequences of migrants are not associated with the dynamics of the epidemic. This would be observed as a non-convergence of health outcomes between migrants and non-migrants over time. In this instance, we would attribute the persisting differential health consequences amongst migrants and non-migrants to structural determinants that are independent of the epidemic dynamics. These may be individual, household or community factors, or factors relating to health and social systems that result in discriminatory barriers to prevention or treatment.

These convergence or non-convergence patterns may apply to both infectious and non-communicable diseases. Vector-borne diseases may spread more easily through physical contacts but behaviours and differential health care also contribute to the spread of diseases whether communicable or not. We would expect that migrants are more subjected to the divergence-convergence cycle in the case of infectious diseases because of the assumed greater role of the physical environment (transmission of viruses, bacteria, and parasites). However the divergence-convergence cycle may also apply to transmission of health behaviours (e.g.: eating and drinking habits) across social environments through migrants. Conversely the divergence-convergence pattern may not apply at all if migration status is unrelated to physical or social propagation. The epidemic dynamics would then be independent of the migration pattern.

## Methods

### Study population

The paper employs data from four HDSSs located in Kenya and South Africa. These HDSS centres are members of the International Network for the Demographic Evaluation of Populations and Their Health (INDEPTH), and are part of the INDEPTH MADIMAH project which uses standardised data formats and protocols to analyse prospective longitudinal data on migration and health see [29–31]. The HDSS method continuously registers all births, deaths and in- and out-migrations within a geographically defined population. The two South African HDSS sites included in the analysis, Agincourt and the Africa Health Research Institute (AHRI), are located in mostly-rural settlement types in two different provinces of the country. The two Kenyan HDSSs are located in Nairobi and Kisumu. The Nairobi surveillance area consists of two non-contiguous, densely populated urban neighbourhoods, while the Kisumu HDSS is a contiguous, mostly-rural settlement type (see Table 1 for characteristics of the HDSS sites in the sample). The four HDSSs were selected from two countries with high levels of premature adult mortality and a high burden of HIV. In South Africa and Kenya, life expectancy at birth was most recently estimated at 59 years and 61 years respectively, while HIV/AIDS was the leading cause of death in both countries with 54.5 deaths per 1000 attributed to this cause in Kenya and 202.1 per 1000 in South Africa [32]. In 2012, the probability of death between the ages 30 and 70 due to four major NCDs (cancer, cardiovascular disease, chronic respiratory disease and diabetes) was estimated at 27% in South Africa, and 18% in Kenya respectively [32]. The HDSSs included in the study met the following criteria for inclusion in the analysis: cause of death data had been collected, there were a sufficient number of deaths by analysis category, and these sites had undertaken a minimum of nine years of follow-up. Nine years of follow-up was used as an inclusion criterion since it provides a minimum duration of time to analyse migrants who had left and then returned to the HDSS area. In a prior analysis, no significant difference in mortality risk amongst migrants and permanent residents was found ten years following migration due to the effect of adaptation, thus migrants who have been in the HDSS areas for ten years or longer are regarded as permanent residents [21].

**Table 1:** HDSS sites included in this multi-centre analysis

HDSS Site	Population Size (approximate)	Size of Site (km^2^)	Settlement Type	Population Density Estimate (persons per km^2^)	Inception Year	Contiguity and Location
SOUTH AFRICA						
Agincourt HDSS South Africa [49]	91,178	420	(Mostly) Rural	217.1	1992	Contiguous site situated in northeast South Africa close to border with Mozambique.
AHRI HDSS South Africa [50]	85,000	438	(Mostly) Rural	194.1	1997	Contiguous site in the Umkanyakude district of KwaZulu-Natal.
KENYA						
Kisumu HDSS Kenya [40]	223,406	700	(Mostly) Rural	319.2	2001	Contiguous site located in Siaya County (HDSS comprises Asembo, Gem and Karemo sub-countries), situated northeast of Lake Victoria, Nyanza Province, Western Kenya.
Nairobi HDSS Kenya [51]	71,000	0.97	Urban	73,195.9	2002	Non-contiguous site comprising Viwandani and Korogocho slum settlements (7 km apart) in capital, Nairobi.

### Variables and analysis

Data on causes of death were collected using verbal autopsies that were conducted according to WHO standards [33, 34]. Cause of death assignments based on verbal autopsy data were computed using InterVA4 ver4.02 [35], with cause of death categories corresponding to International Classification of Diseases (ICD 10) [36, 37]. These methods produce standardised data on cause of death across the study locations. In the analysis, causes of death were grouped into a set of broad categories: major risk infectious disease (HIV/AIDS related death or pulmonary tuberculosis); other infections (e.g.: acute respiratory infections, malaria); NCDs (e.g.: diabetes mellitus, acute cardiac disease, stroke) and neoplasms; maternal and neonatal causes; external causes (e.g.: road traffic accidents, assault, self-harm), and unknown or indeterminate causes. The focus of the study is on AIDS/TB and NCD mortality because of the interest in disease dynamics of these two dominant epidemics. Nevertheless, probability of death by cause is presented for all cause categories.

**Fig. 1:**
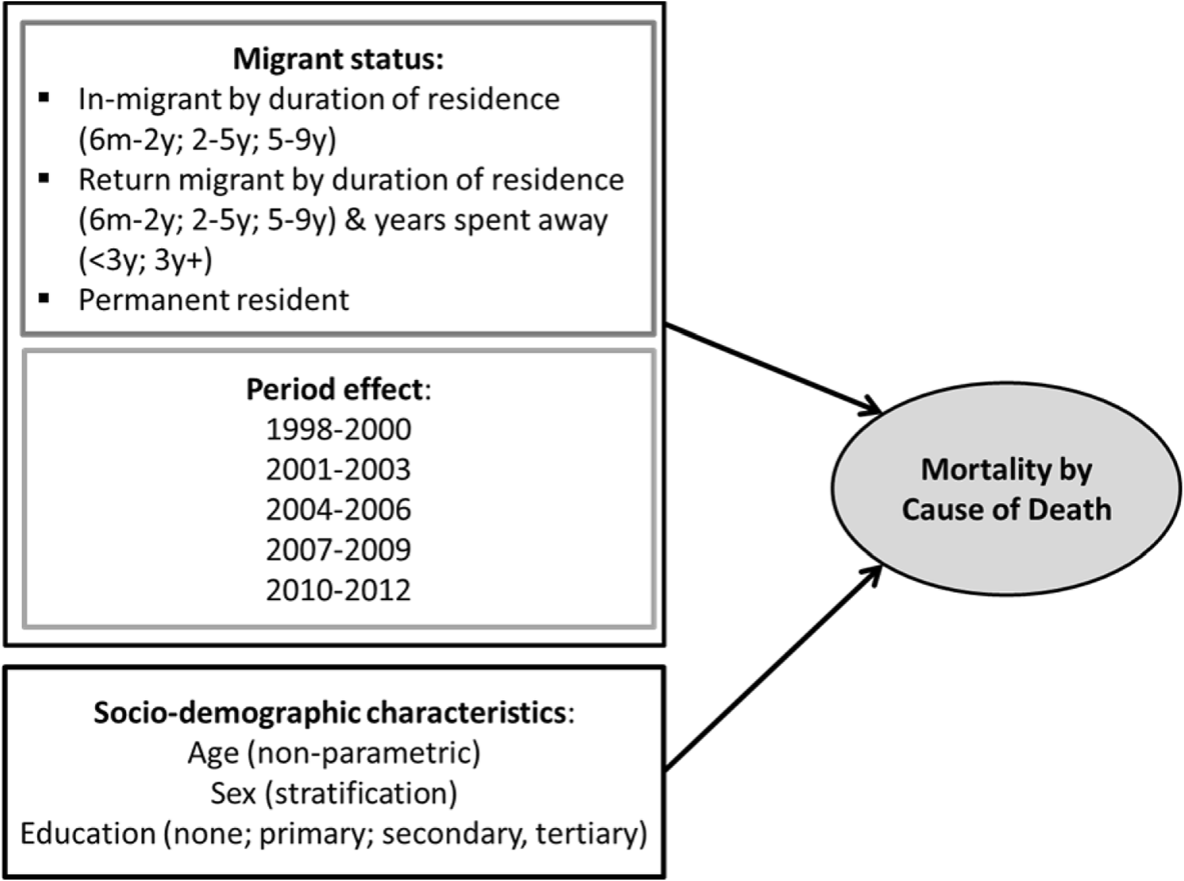
Migration-Death Competing Risk Model

The migration-death competing risk conceptual model employed in the analysis is presented in Fig. 1. Migration in this analysis is defined as a move that crosses the geographical boundary of the HDSS site in an inward or outward direction. Moves that take place within an HDSS area are therefore excluded from the analysis. HDSS sites may apply different time thresholds to define an in- or out-migration, ranging from three to six months residency following a move. In order to standardise the migration definition across the HDSS sites in the study, a six-month residency threshold was applied to determine an individual’s residency status in the surveillance area (see [21, 31] for more details on migration methods).

Migration status is defined as either first time in-migrant to the HDSS area, return migrant to the HDSS or permanent resident (individuals who have not migrated). In-migrants are individuals who have not previously resided in the HDSS surveillance area, while return migrants are former residents who have temporarily relocated (generally to take up employment). In the case of in- and return migrants, the analysis further discriminates risk of mortality by the duration of time since entry into the HDSS area. The effect of duration was observed to be significant in previous work [21]. The models used in this study control for three categories of duration following an in- or return migration to the HDSS area: six months to two years; two to five years and five to nine years. For in-migrants and return migrants, the reference category employed in the models represents the most recent migrants (migrants who have been in the HDSS area for between six months and two years). Migrants who have been in the HDSS areas for ten years or longer are regarded as permanent residents, while very recent in-migrants and return migrants are excluded from the analysis due to the six-month residency threshold described above. For return migrants, the length of time spent outside the HDSS is also controlled for in the models to represent the migrant’s exposure to the destination area prior to return home (with a longer duration of more than three years contrasted with a shorter duration of less than three years). The models therefore control for a net effect of duration of residence, as well as duration of exposure outside the HDSS.

The analysis controls for calendar effects in order to capture the dynamics of the AIDS/TB and NCDs epidemics. Time is divided into three-year periods, which ensures that trends in mortality over time are captured for the different diseases and short-term fluctuations reduced. The four HDSS sites contribute different periods to the analysis depending on the length of time since inception. All sites contribute data from the year 2004 onwards. Models include an effect of period where its coefficients represent the average period effect for permanent residents.

In order to isolate a migration effect by period, the models control for the interaction between period effect and migration status. These terms represent in-migrants and return migrants in the respective periods who have resided in the HDSS area for between six months and two years following entry. The reference category represents permanent residents for each corresponding period. These terms allow us to test the hypothesis of convergence of in-migrants and return-migrants to that of permanent residents in the population over the observation period. Convergence is observed for a specific disease if the difference in mortality risk between migrants and non-migrants declines over time, implying that migration status has become less relevant to the dynamics of the disease. Conversely, the difference in mortality risk by migrant status can increase over time (diverge), or remain stable, showing no relationship to the epidemic.

All analyses are performed separately for males and females because of the difference in migration and mortality patterns between the sexes. Finally, the models control for the following sociodemographic characteristics: age (limited to a 15–60 year age range in accordance with the definition of premature adult mortality) and education level which is time-varying (standardised across the four sites to contrast no formal education with primary, secondary and tertiary-level education).

The Fine and Gray statistical model is used for estimation and is based on the cumulative incidence function that does not assume independence of cause of death [38]. This is a superior approach to the regular Cox proportional hazards model which makes the assumption of independence of different causes of death in the analysis of mortality. The use of this method showed only slight differences in the results compared with the method based on simple hazard rates.

The statistical model for each large cause of death, and separately for males and females, may be written as follows:

$$ {\displaystyle \begin{array}{c} logH\left[t,X(t)\right]={logH}_0(t)+{\beta}_1\ast period\\ {}+{\beta}_2\ast inmigrantperiod+{\beta}_2\ast retmigrantperiod\\ {}+{\alpha}_1\ast inmigrantduration+{\alpha}_2\ast retmigrantduration\\ {}+\gamma \ast retmigrantexposure+\delta \ast educationlevel\end{array}} $$

Where:

- “period” is a set of indicators defining each 3-year period (2010–2012 is the reference category),
- “inmigrantperiod” and “retmigrantperiod” are two sets of indicators defining periods for in-migrants and return migrants respectively; to note, their effects are measured net of, and add on to the main effect of period,
- “inmigrantduration” and “retmigrantduration” are two sets of indicators defining duration since migration into the HDSS for in-migrants and return migrants respectively (with reference category “6 months to 2 years”),
- “retmigrantexposure” is an indicator defining the duration spent outside the HDSS for return migrants only (with reference category less than or equal to 3 years),
- “educationlevel” is an indicator defining the individual’s broad level of education (the reference category is no formal education)

H_0_ is the baseline cumulative incidence function for all the above indicators set to zero (i.e.: representing mortality over the 2010–2012 period for non-migrants with no formal education).

## Results

### Descriptive results

The characteristics of the sample are presented as an additional table (see Additional file 1: Table S1), which details the person years for all variables in the analysis, by sex. All variables included in the models have person-years at risk (PYAR) in excess of 500. Additional file 1: Table S1 indicates that in- and return-migrants in their first ten years of residence represent between 59% (females in Nairobi) and 27% (males in Kisumu) of the total PYAR during the study period. Rates of in- and out-migration in these HDSS sites were reported on in an earlier publication see [31]. The number and percentage of adult deaths due to AIDS/TB and NCDs, by HDSS site and sex are presented in Additional file 2: Table S2. In all sites, AIDS/TB accounts for the largest proportion of total deaths between ages 15 and 60 (ranging from 36% of deaths among males in the Nairobi HDSS to 71% of deaths among females in the AHRI HDSS).

**Fig. 2:**
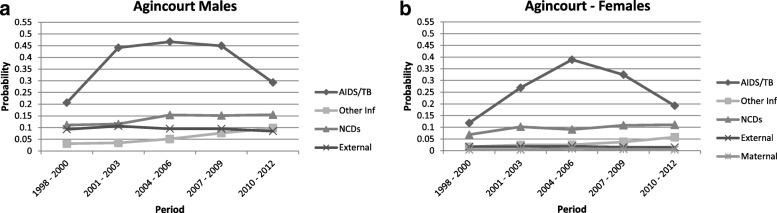
Probability of death between ages 15 and 60 by cause category: Agincourt

**Fig. 3:**
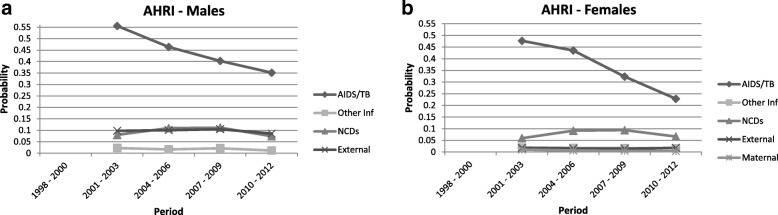
Probability of death between ages 15 and 60 by cause category: Africa Health Research Institute

**Fig. 4:**
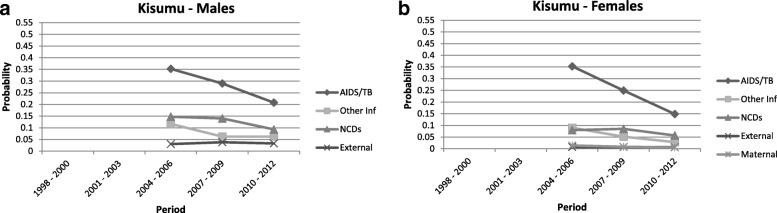
Probability of death between ages 15 and 60 by cause category: Kisumu

**Fig. 5:**
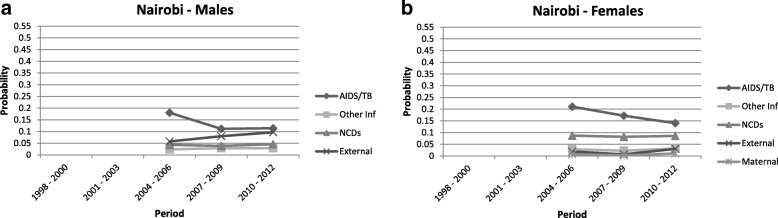
Probability of death between ages 15 and 60 by cause category: Nairobi

The probability of death by cause amongst adults aged between 15 and 60 years (i.e.: 45q15, noted “q” for simplicity in the following) is presented in Figs. 2, 3, 4 and 5. These probabilities are calculated based on the cumulative incidence function meaning that they take into account the competing risk. These graphs depict the transition in mortality for each cause of death over the period of analysis. Note that across the four HDSS sites, indeterminate and unknown causes of death comprised between 5 and 15% of all deaths.

#### Agincourt HDSS, South Africa

The length of mortality monitoring in the Agincourt HDSS captures the rise and fall of the AIDS/TB epidemic in this area. Amongst males and females in the Agincourt HDSS, mortality due to AIDS/TB increased in the early 2000s and, following an inverse-U shape, began to decline in 2006 with the roll-out of antiretroviral therapy (ART). Males experienced slightly higher mortality due to AIDS/TB as compared with females (in the 2010–2012 period q = 0.29 for males and q = 0.19 for females). The probability of mortality due to NCDs began to increase from 2004 for males, reaching 0.16 in the most recent period, and is gradually increasing amongst females (q = 0.11 in 2010–2012). The two main causes of death display different trends, one decreasing and the other increasing.

#### Africa Health Research Institute HDSS, South Africa

The rapid rise in AIDS/TB-related mortality in the AHRI HDSS is not captured on the graph due to population surveillance starting in the year 2000 after the epidemic was well established. The charts do however capture the decline in AIDS/TB mortality. The probability of death due to AIDS/TB is slightly lower amongst females in the 2010–2012 period (q = 0.23) as compared with males (q = 0.35). With regards to NCDs, there was a slight increase until 2009, but the probability of premature adult mortality due to NCDs decreased in the recent period (q = 0.07 for both males and females).

In both the AHRI and Agincourt HDSS populations, high levels of external causes of death are apparent amongst males (around q = 0.10).

#### Kisumu HDSS, Kenya

Over the nine years of data available for Kisumu, the decline of the AIDS/TB epidemic is apparent as the probability of death amongst males and females decreased to q = 0.21 and q = 0.15 in the 2010–2012 period respectively. The probability of death from NCDs for both sexes in Kisumu decreased in this same period (q = 0.09 for males and q = 0.06 for females).

#### Nairobi HDSS, Kenya

The nine years of data from the Nairobi HDSS indicate lower AIDS/TB mortality as compared to the other three sites, albeit higher amongst females (q = 0.11 for males in the 2010–2012 period, and q = 0.14 for females). In these urban neighbourhoods, the trends in NCD mortality have remained stable over the time frame, but higher amongst females (for males q ≈ 0.04 and females q ≈ 0.09). To note that the Nairobi HDSS experienced an upward trend in external deaths amongst males (q = 0.10) in 2010–2012.

In summary, the trends with respect to the four HDSS sites suggest similarities between the sexes within each site. Nevertheless, in cases where sex differences are observed, males tend to experience higher levels of premature adult mortality as compared with females (the exception being in the case of AIDS/TB and NCD deaths in Nairobi).

### Results of the competing risk models

**Fig. 6:**
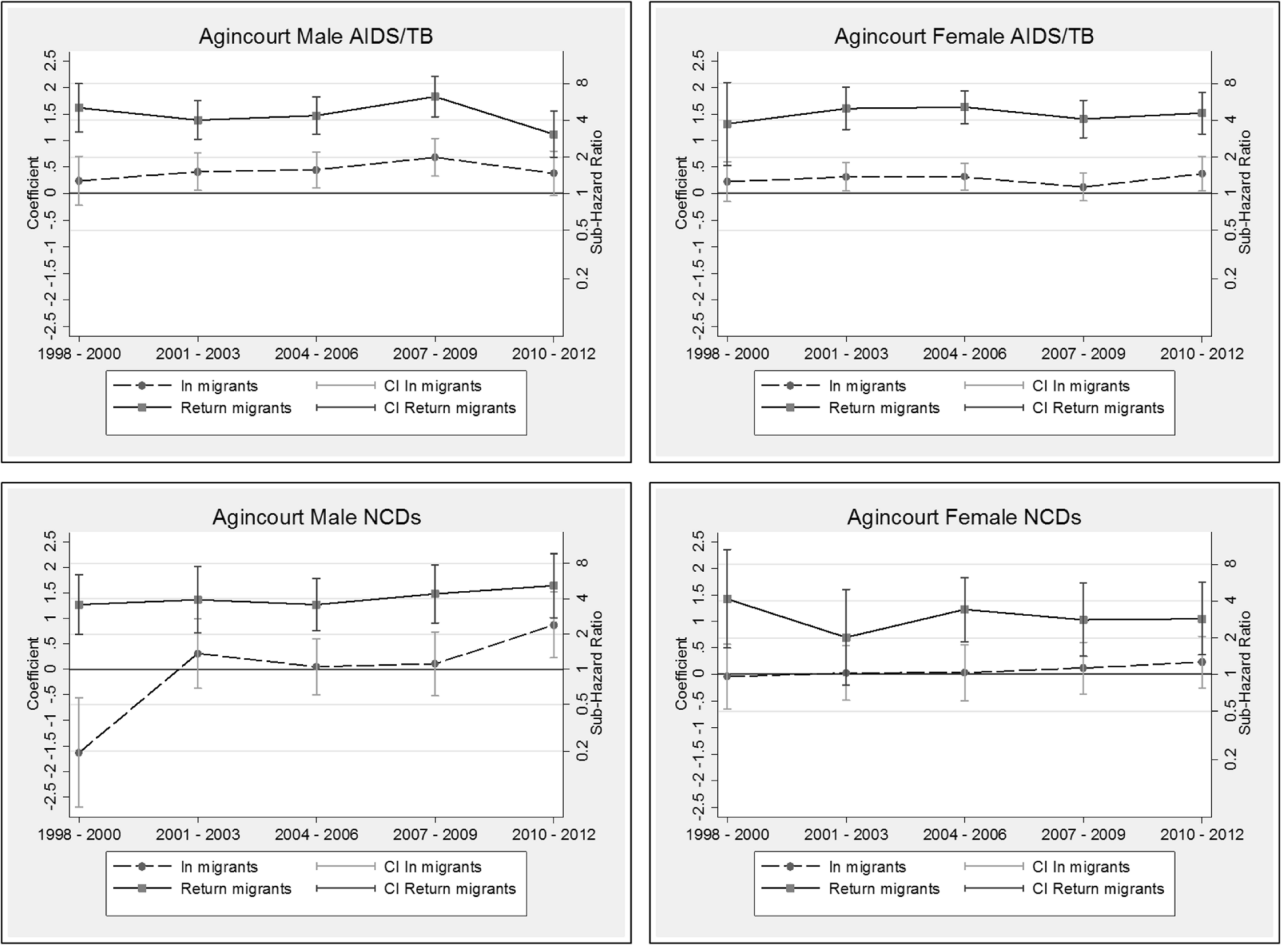
Agincourt HDSS competing risk models

**Fig. 7:**
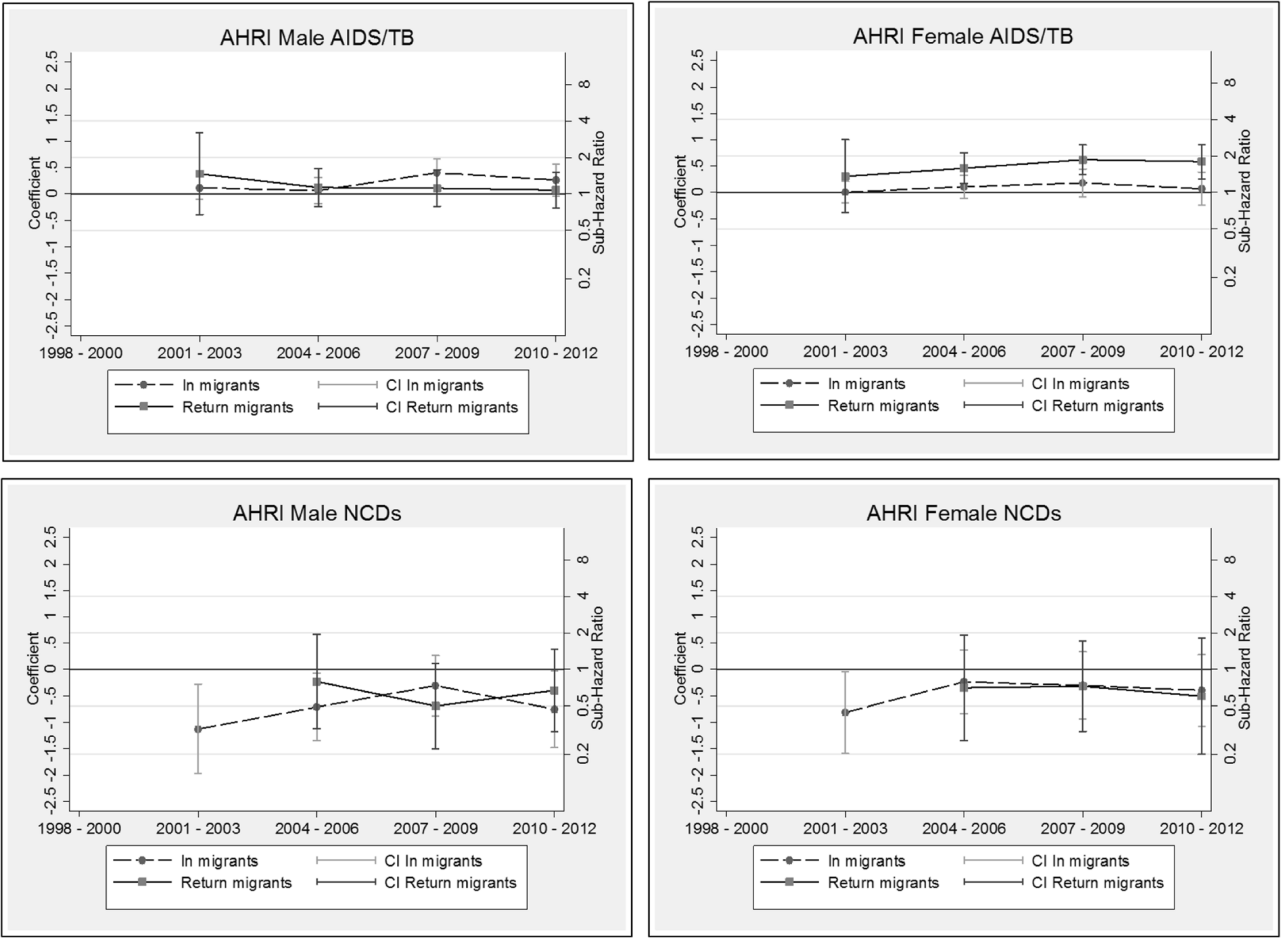
Africa Health Research Institute HDSS competing risk models

**Fig. 8:**
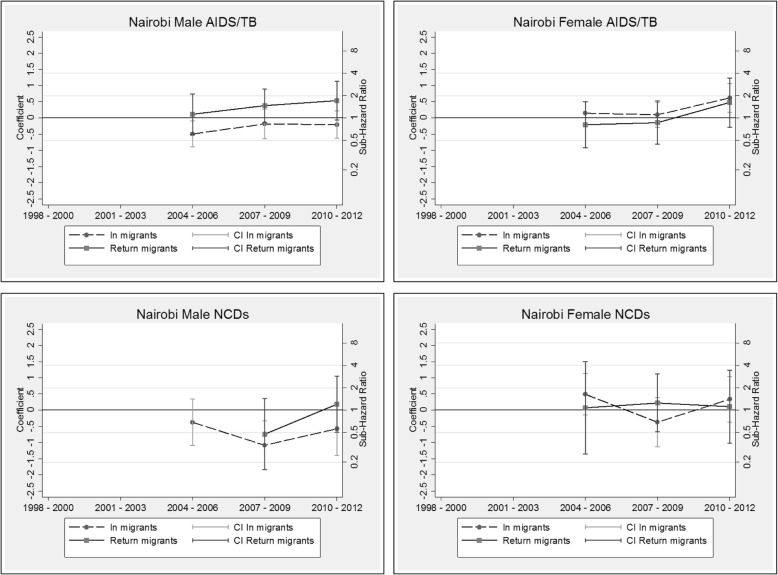
Kisumu HDSS competing risk models

**Fig. 9:**
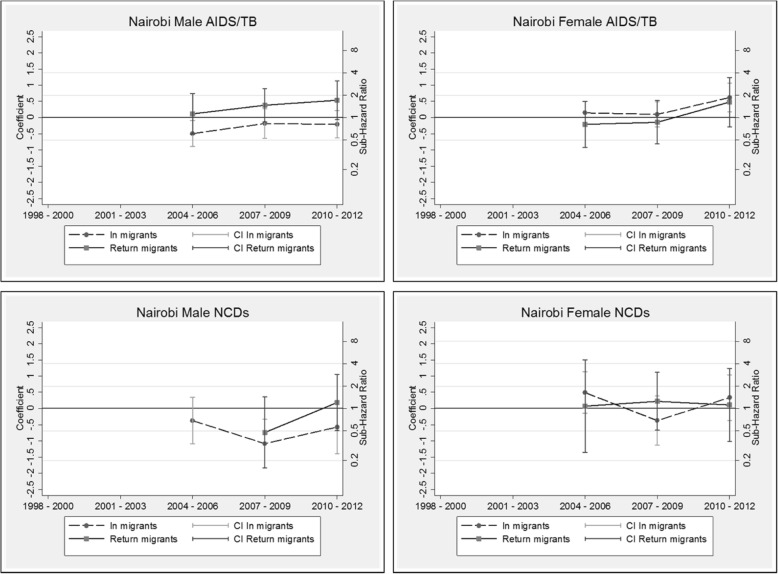
Nairobi HDSS competing risk models

The competing risk models are presented as additional tables (see Additional file 3: Table S3, Additional file 4: Table S4, Additional file 5: Table S5 and Additional file 6: Table S6). The coefficients by migration status and period (and corresponding sub-hazard ratios on the right-hand scale) are displayed graphically by HDSS site, cause and sex in Figs. 6, 7, 8 and 9. These graphs allow us to examine how migration status contributes to the trends in mortality over time, after controlling for calendar and duration of residence (adaptation) effects. In all sites, the models reveal a decline in the risk of mortality with increasing duration of residence since entry into the HDSS area. The in-migrants and return migrants represented are those who have resided in the respective HDSS areas for between six months and two years following migration (the reference category in the models). For return migrants, the trends represent those who spent less than three years outside of the HDSS (the reference category). In each graph, permanent residents are represented by the value zero on the coefficient axis and the value one on the sub-hazard ratio axis. Where the graphs by migration status present as a horizontal line, it indicates that the effect of migration status has not changed over time of (i.e.: the period trends observed in Figs. 2, 3, 4 and 5 hold for the full population, regardless of migration status). Else, the graphs may reveal a pattern of convergence or divergence between migrants and non-migrants.

#### Agincourt HDSS, South Africa

The competing risk model for Agincourt reveals significant differences in the risk of mortality due to AIDS/TB between return migrants, in-migrants and permanent residents. On average, return migrants to the HDSS are four times more likely to die of AIDS/TB than are permanent residents. This trend is fairly stable over time (the 95% confidence intervals show little difference from one period to another), with the trend amongst females being even more stable than that of males. This consistency over time is independent of calendar and duration effects controlled by other variables (see Additional file 3: Table S3). These findings confirm the phenomenon of migrants “returning home to die”, and indicate that this is not transitionary – there is no convergence with non-migrants over time. On average, the in-migrants to Agincourt have a slightly higher risk of mortality as compared with permanent residents for both males and females.

In the case of NCDs, return migrants present with very similar risks of mortality relative to non-migrants, as was the case for AIDS/TB. The risk for males dying of NCDs is on average four times higher than that of permanent residents, and for females approximately three times higher. In-migrant females do not display a significantly different risk profile for NCD mortality compared to non-migrants in the HDSS area. For males, it appears that the risk amongst in-migrants is increasing in more recent periods but this trend would need to be confirmed with data on subsequent periods.

#### Africa Health Research Institute HDSS, South Africa

In contrast with the Agincourt results, AIDS/TB mortality amongst AHRI migrant males does not differ significantly from that of permanent residents. However, female return migrants to the AHRI HDSS have approximately twice the risk of dying from AIDS/TB from the year 2004 onwards, as compared with permanent residents in the population. These trends by migrant status are fairly stable over time. With regards to NCD mortality, both male and female migrants seem somewhat protected as compared with permanent residents. However, the difference in migrant status is not statistically significant. Overall, the risk profile for migrants as compared to non-migrants with regards to AIDS/TB and NCD mortality is lower in the AHRI study site, in comparison with Agincourt.

#### Kisumu HDSS, Kenya

In the Kisumu HDSS, the mortality profile does not differ significantly between first time in-migrants to the area and returnees. Both categories of migrant have a slightly higher risk of AIDS/TB mortality as compared with permanent residents, with females having a higher risk than males. Similarly, for NCD mortality, female migrants of both categories have a higher risk than their male migrant counterparts, as compared to non-migrants.

#### Nairobi HDSS, Kenya

The Nairobi HDSS models reveal that female return migrants and in-migrants do not differ significantly with respect to AIDS/TB mortality risk, while the risk of death from AIDS/TB amongst males returning to the urban area is higher than for first time in-migrants. For female migrants from 2010 onwards, there is some divergence in AIDS/TB mortality risk from that of permanent residents who have lower risk. Data on subsequent periods would shed light on this trend. For NCD mortality, there is no apparent difference between in-migrants and return migrants for both males and females.

## Discussion

This is the first study to analyse the extent to which internal migrant status is associated with patterns of mortality by cause of death. The results of the study shed light on both the dynamics of the AIDS/TB and NCD epidemics underway in these South African and Kenyan local areas, and the extent to which the mortality of migrants converges to that of non-migrants as the epidemic evolves. In all four HDSS populations, AIDS/TB accounts for a significant proportion of total deaths. In the Agincourt HDSS, there is evidence of a gradual increasing trend in NCD mortality. In general, the migration effect on mortality over the period of observation presents similar patterns in relation to both infectious and non-communicable diseases, and shows a migrant mortality disadvantage and no convergence in mortality risk between migrants and non-migrants.

In the Agincourt HDSS no convergence or divergence by migrant status is observed – either in relation to AIDS/TB or NCD mortality. In-migrants and even more so, return migrants have a persistent health disadvantage, which has been corroborated in previous studies [12, 21]. This holds for both males and females, and is independent of the diffusion of diseases. In-migrants to the Agincourt HDSS area generally originate from surrounding rural areas that carry similar health risks [13, 39]. Return migrants most commonly move to metropolitan areas or secondary cities to access employment, and may return home with physical or mental health issues. The higher mortality risk among return migrants is of particular concern in this population.

In the Kisumu HDSS, the main features of the migration-mortality relationship are the lack of significant differences between in-migrants and return migrants, and the greater risk of AIDS/TB mortality amongst female migrants. One hypothesis for the similarity between in-migrants and return migrants is that their exposure to health risks is similar within and outside the HDSS area. Although the Kisumu HDSS is built upon a strong health provision infrastructure run by the Ministry of Health and its collaborative partners, which emphasises ART and malaria treatment [40], research has suggested that mobile females from the Kisumu area who are HIV positive may experience interrupted ART as a result of access issues and mobility [41].

In two instances, the trends by migration status are less stable over time. This is indicated by an increase in AIDS/TB mortality risk amongst females from the AHRI HDSS from 2004, and a divergence in AIDS/TB mortality risk amongst Nairobi females in the most recent period. It is well established that female return migrants to the AHRI HDSS face health challenges as they are significantly more likely to die, particularly from HIV-related conditions [42], as compared with residents of both sexes [43]. Poor linkage to regular ART may help to explain the higher AIDS/TB-related mortality amongst female migrants. A number of studies originating from the ARHI study site point to proximity to primary care, and gender inequality as major barriers to accessing care among this vulnerable population [44]. Female return migrants who spend more time away from the AHRI study site have been observed to have a greater likelihood of HIV acquisition compared with males [45].

In the Nairobi HDSS, AIDS/TB and NCD mortality is higher amongst females as compared with males, while external causes of death are consistent with the expected (i.e.: males are at higher risk). This corroborates a study by Mberu et al. [46] that found a number of gender variations in causes of death in the Nairobi HDSS that were inconsistent with the literature. For cardiovascular diseases, Mberu et al. [46] suggest that misperception by health practitioners may lead to underestimated risk and under-diagnosis amongst females, resulting in higher mortality. They may be more vulnerable and often stigmatised, with less economic opportunities to sustain themselves and their children [47]. Both married and single mothers are often tied down to a particular location in the slums and are therefore less mobile [48]. Thus for females in Nairobi, the observed patterns may be explained by conditions and circumstances of migrants in the urban areas. The hypothesis is that in case of an adverse health event, females are less likely than males to leave their households in the slums to seek treatment or care. However, the complexity of risk and selection factors is likely to contribute to the instability of the relationship between migration status and mortality in Nairobi.

HDSSs provide a versatile platform to study a diverse range of settings, and offer detailed measures of migration and mortality dynamics. The value of a comparative perspective is that it highlights the differences but also the commonalities in the relationship between internal migration status and changing disease patterns in these local areas. A study limitation is that the analysis does not include information about reasons for movement or specific migrant destinations. Further detail on social and economic conditions that would explain the differences in mortality between migrants and non-migrants would add value. These more detailed contextual dimensions are being explored as part of site-specific studies aimed at collecting detailed survey data by following migrants who leave the HDSS areas.

## Conclusion

In conclusion, the study findings do not confirm the hypothesis of mortality convergence between migrants and non-migrants. There is no apparent association between migration status and epidemic dynamics as no period convergence by migrant status is observed. A convergence (or divergence) would indicate the reduction (or increase) in adverse health conditions for migrants. Rather, the stability by migration status over time and over a range of settings, for both infectious diseases and NCDs, is suggestive of a general health care deficit for migrants. Findings suggest that structural issues rather than epidemic dynamics explain difference in mortality risk by migrant status. Factors such as poor access to health care at destinations, poor social integration, inadequate living conditions at destinations, employment status, stress experienced as a result of relocation, or behavioural factors associated with migration may all contribute to these observed differences by migration status. The findings also suggest that female migrants may be particularly vulnerable in certain contexts.

Despite the differences observed across sites, a similar policy message may apply. Recent migrants should be identified and targeted by the health systems to improve their access to treatment at all stages of the epidemic, whether infectious or non-infectious. Interventions aimed at educating people who intend leaving an area may be an effective means of mitigating potential risks at migrants’ destinations. Further research into the circumstances at migrant destinations in these various contexts is a priority going forward.

## Additional files

Additional file 1: Table of person years by HDSS site and sex. (DOCX 34 kb)

Additional file 2: Number and percentage of AIDS/TB and NCD deaths by HDSS site and sex. (DOCX 27 kb)

Additional file 3: Agincourt HDSS Competing Risk Models. (DOCX 31 kb)

Additional file 4: Africa Health Research Institute HDSS Competing Risk Models. (DOCX 30 kb)

Additional file 5: Kisumu HDSS Competing Risk Models. (DOCX 29 kb)

Additional file 6: Nairobi HDSS Competing Risk Models. (DOCX 30 kb)

## Abbreviations

45q15: Probability of death among adults aged between 15 and 60 years
AHRI: Africa Health Research Institute
AIDS: Acquired immune deficiency syndrome
ART: Antiretroviral therapy
HDSS: Health and Demographic Surveillance System
HIV: Human immunodeficiency virus
ICD: International Classification of Diseases
INDEPTH: International Network for the Demographic Evaluation of Populations and their Health
MADIMAH: Multi-centre Analysis of the Dynamics of Internal Migration And Health
NCD: Non-communicable disease
PYAR: Person-years at risk
TB: Tuberculosis
WHO: World Health Organization
