# Correction to: association between internal migration and epidemic dynamics: an analysis of cause-specific mortality in Kenya and South Africa using health and demographic surveillance data

**DOI:** 10.1186/s12889-021-11604-z

**Published:** 2021-08-16

**Authors:** Carren Ginsburg, Philippe Bocquier, Donatien Béguy, Sulaimon Afolabi, Kathleen Kahn, David Obor, Frank Tanser, Andrew Tomita, Marylene Wamukoya, Mark A. Collinson

**Affiliations:** 1 Medical Research Council/Wits Rural Public Health and Health Transitions Research Unit (Agincourt), School of Public Health, Faculty of Health Sciences, University of the Witwatersrand, 27 St Andrews Road, Parktown, Johannesburg, 2193 South Africa; 2 INDEPTH Network, Accra, Ghana; 3 Centre de Recherches en Démographie, Université Catholique de Louvain, Louvain-la-Neuve, Belgium; 4 African Population and Health Research Centre, Nairobi, Kenya; 5 Umeå Centre for Global Health Research, Umeå University, Umeå, Sweden; 6 KEMRI & CDC - Centre for Global Health Research, Kisumu, Kenya; 7 Africa Health Research Institute, University of KwaZulu-Natal, Durban, South Africa; 8 Centre for the AIDS Programme of Research in South Africa (CAPRISA), Durban, South Africa; 9 School of Nursing and Public Health, University of KwaZulu-Natal, Durban, South Africa; 10 Nelson R Mandela School of Medicine, University of KwaZulu-Natal, Durban, South Africa; 11 Department of Science and Technology/ Medical Research Council, South African Population Research Infrastructure Network, Johannesburg, South Africa

**Correction to: BMC Public Health 18, 918 (2018)**

**https://doi.org/10.1186/s12889-018-5851-5**

Following publication of the original article [1], the co-authors observed that Figure 8, although titled correctly, contained the same set of graphs as Figure 9. The correct Figure 8, depicting the competing risk models for the Kisumu HDSS, is presented below:

**Fig. 8:**
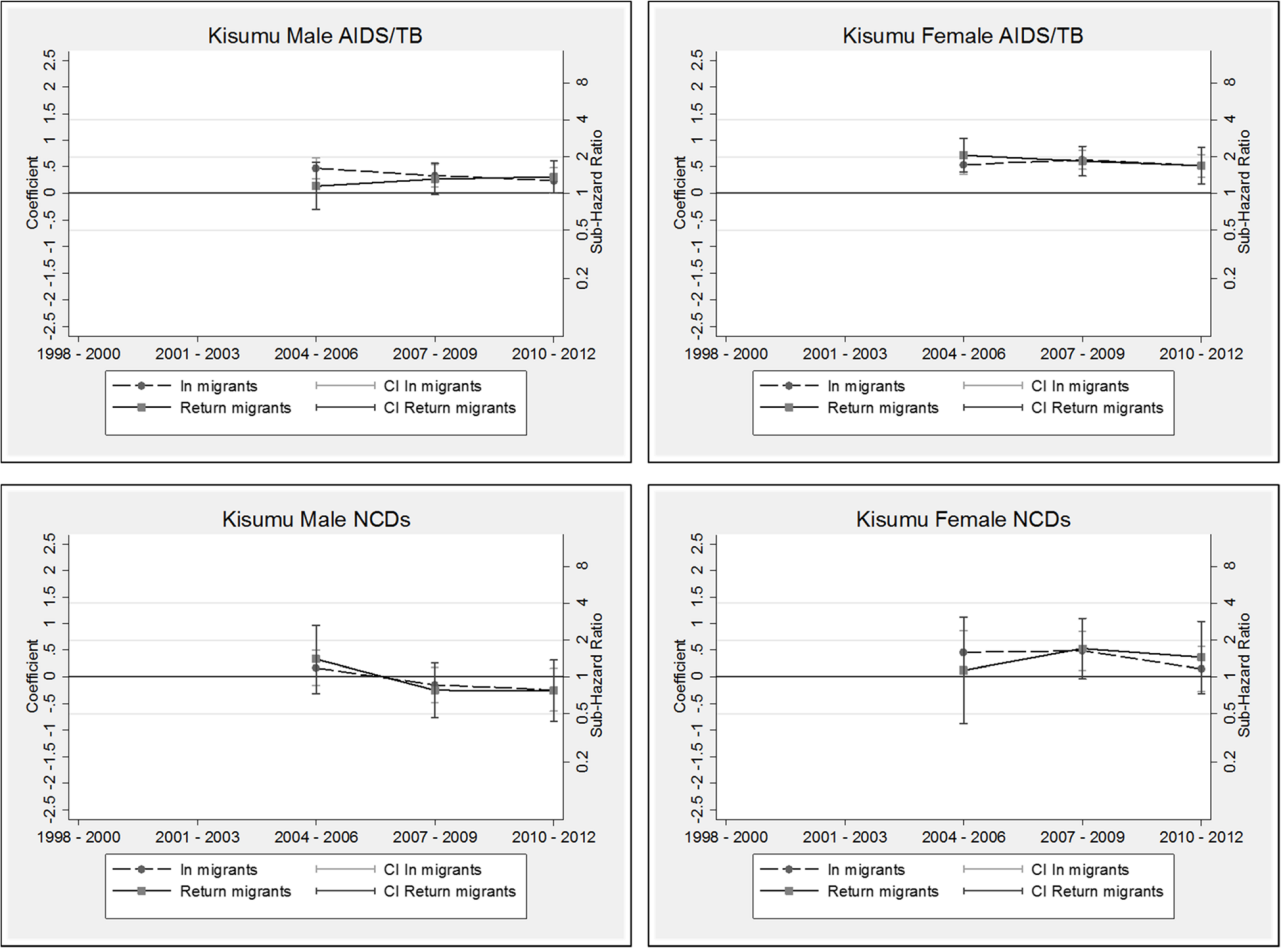
Kisumu HDSS competing risk models

## References

[1] Ginsburg (2018). Association between internal migration and epidemic dynamics: an analysis of cause-specific mortality in Kenya and South Africa using health and demographic surveillance data. BMC Public Health.

