# Supplementary material for: Association between internal migration and epidemic dynamics: an analysis of cause-specific mortality in Kenya and South Africa using health and demographic surveillance data
Source: BMC Public Health. 2018 Jul 27;18:918. doi: 10.1186/s12889-018-5851-5 (PMC6062880; doi:10.1186/s12889-018-5851-5)
Supplement: Supplementary file 1 — Table of person years by HDSS site and sex. (DOCX 34 kb) [file 12889_2018_5851_MOESM1_ESM.docx]

**Table S1: Table of person years by HDSS site and sex**

|  | **Agincourt HDSS** | | **AHRI HDSS** | | **Kisumu HDSS** | | **Nairobi HDSS** | |
| --- | --- | --- | --- | --- | --- | --- | --- | --- |
|  | Male | Female | Male | Female | Male | Female | Male | Female |
| **Total Person Years** | 166 915 | 246 816 | 167 606 | 224 723 | 354 672 | 425 264 | 192 833 | 134 336 |
|  | 100% | 100% | 100% | 100% | 100% | 100% | 100% | 100% |
| **Non-migrant** | 93 551 | 125 691 | 109 304 | 149 530 | 259 193 | 304 621 | 83 473 | 54 691 |
|  | 56% | 51% | 65% | 67% | 73% | 72% | 43% | 41% |
| **In-migrant** | 59 399 | 109 805 | 37 458 | 49 812 | 63 390 | 88 512 | 89 428 | 64 961 |
|  | 36% | 44% | 22% | 22% | 18% | 21% | 46% | 48% |
| **Return migrant** | 13 965 | 11 320 | 20 844 | 25 381 | 32 089 | 32 131 | 19 932 | 14 684 |
|  | 8% | 5% | 12% | 11% | 9% | 8% | 10% | 11% |
| **Duration since  in-migration** |  |  |  |  |  |  |  |  |
| 6m-2y in-migrant | 15 234 | 30 238 | 12 790 | 16 088 | 25 949 | 38 788 | 43 528 | 32 213 |
|  | 9% | 12% | 8% | 7% | 7% | 9% | 23% | 24% |
| 2-5y in-migrant | 20 974 | 40 281 | 14 140 | 18 834 | 25 986 | 35 284 | 33 923 | 24 105 |
|  | 13% | 16% | 8% | 8% | 7% | 8% | 18% | 18% |
| 5-9y in-migrant | 23 191 | 39 286 | 10 528 | 14 890 | 11 455 | 14 440 | 11 977 | 8 643 |
|  | 14% | 16% | 6% | 7% | 3% | 3% | 6% | 6% |
| **Duration since  return migration** |  |  |  |  |  |  |  |  |
| 6m-2y in-migrant | 6 021 | 4 458 | 9 156 | 10 472 | 14 938 | 15 541 | 8 883 | 6 605 |
|  | 4% | 2% | 5% | 5% | 4% | 4% | 5% | 5% |
| 2-5y return migrant | 5 050 | 4 207 | 8 244 | 10 221 | 13 369 | 12 945 | 8 192 | 5 950 |
|  | 3% | 2% | 5% | 5% | 4% | 3% | 4% | 4% |
| 5-9y return migrant | 2 894 | 2 655 | 3 444 | 4 688 | 3 782 | 3 645 | 2 857 | 2 129 |
|  | 2% | 1% | 2% | 2% | 1% | 1% | 1% | 2% |
| **Return Migrant Exposure** |  |  |  |  |  |  |  |  |
| <36months | 5 457 | 5 744 | 15 960 | 19 922 | 26 924 | 27 509 | 17 678 | 13 221 |
|  | 3% | 2% | 10% | 9% | 8% | 6% | 9% | 10% |
| 36+ months away | 8 508 | 5 576 | 4 884 | 5 459 | 5 165 | 4 622 | 2 254 | 1 463 |
|  | 5% | 2% | 3% | 2% | 1% | 1% | 1% | 1% |
| **Period (for perm residents only)** |  |  |  |  |  |  |  |  |
| 1998 – 2000 | 23 137 | 28 739 |  |  |  |  |  |  |
|  | 14% | 12% |  |  |  |  |  |  |
| 2001 – 2003 | 16 626 | 23 107 | 34 517 | 45 861 |  |  |  |  |
|  | 10% | 9% | 21% | 20% |  |  |  |  |
| 2004 – 2006 | 16 783 | 23 487 | 27 627 | 37 833 | 69 269 | 82 551 | 37 626 | 23 566 |
|  | 10% | 10% | 16% | 17% | 20% | 19% | 20% | 18% |
| 2007 – 2009 | 17 800 | 24 419 | 24 364 | 34 147 | 96 350 | 113 258 | 25 745 | 17 009 |
|  | 11% | 10% | 15% | 15% | 27% | 27% | 13% | 13% |
| 2010 - 2012 (Ref) | 19 205 | 25 939 | 22 796 | 31 689 | 93 574 | 108 812 | 20 102 | 14 116 |
|  | 12% | 11% | 14% | 14% | 26% | 26% | 10% | 11% |

**Table S1: Table of person years (cont’d)**

|  | **Agincourt HDSS** | | **AHRI HDSS** | | **Kisumu HDSS** | | **Nairobi HDSS** | |
| --- | --- | --- | --- | --- | --- | --- | --- | --- |
|  | Male | Female | Male | Female | Male | Female | Male | Female |
| **Migrant status 1998 - 2000** |  |  |  |  |  |  |  |  |
| In-migrant | 12 289 | 20 305 |  |  |  |  |  |  |
|  | 7% | 8% |  |  |  |  |  |  |
| Return migrant | 1 276 | 591 |  |  |  |  |  |  |
|  | 1% | 0% |  |  |  |  |  |  |
| **Migrant status 2001 - 2003** |  |  |  |  |  |  |  |  |
| In-migrant | 11 836 | 22 210 | 5 837 | 8 166 |  |  |  |  |
|  | 7% | 9% | 3% | 4% |  |  |  |  |
| Return migrant | 1 721 | 1 122 | 366 | 443 |  |  |  |  |
|  | 1% | 0% | 0% | 0% |  |  |  |  |
| **Migrant status 2004 - 2006** |  |  |  |  |  |  |  |  |
| In-migrant | 10 966 | 21 286 | 9 752 | 13 219 | 11 596 | 14 755 | 18 360 | 13 583 |
|  | 7% | 9% | 6% | 6% | 3% | 3% | 10% | 10% |
| Return migrant | 2 328 | 2 190 | 2 982 | 3 787 | 2 483 | 2 734 | 2 883 | 2 110 |
|  | 1% | 1% | 2% | 2% | 1% | 1% | 1% | 2% |
| **Migrant status 2007 - 2009** |  |  |  |  |  |  |  |  |
| In-migrant | 11 549 | 22 553 | 11 086 | 14 799 | 20 971 | 28 744 | 30 718 | 22 166 |
|  | 7% | 9% | 7% | 7% | 6% | 7% | 16% | 17% |
| Return migrant | 3 280 | 3 094 | 6 700 | 8 539 | 10 239 | 10 258 | 7 118 | 5 191 |
|  | 2% | 1% | 4% | 4% | 3% | 2% | 4% | 4% |
| **Migrant status 2010 - 2012** |  |  |  |  |  |  |  |  |
| In-migrant | 12 759 | 23 451 | 10 783 | 13 628 | 30 823 | 45 013 | 40 350 | 29 212 |
|  | 8% | 10% | 6% | 6% | 9% | 11% | 21% | 22% |
| Return migrant | 5 360 | 4 323 | 10 796 | 12 612 | 19 367 | 19 139 | 9 931 | 7 383 |
|  | 3% | 2% | 6% | 6% | 5% | 5% | 5% | 6% |
| **Education** |  |  |  |  |  |  |  |  |
| No Formal | 12 098 | 35 814 | 5 283 | 10 209 | 3 834 | 25 148 | 5 166 | 7 902 |
|  | 7% | 15% | 3% | 5% | 1% | 6% | 3% | 6% |
| Some Primary | 34 316 | 44 628 | 19 215 | 35 896 | 219 149 | 278 687 | 107 403 | 85 221 |
|  | 21% | 18% | 11% | 16% | 62% | 66% | 56% | 63% |
| Some Secondary | 110 848 | 150 353 | 96 362 | 122 024 | 85 705 | 71 261 | 76 054 | 39 330 |
|  | 66% | 61% | 57% | 54% | 24% | 17% | 39% | 29% |
| Some Tertiary | 8 140 | 13 912 | 44 650 | 54 317 | 14 525 | 8 204 | 2 708 | 1 037 |
|  | 5% | 6% | 27% | 24% | 4% | 2% | 1% | 1% |
| Unknown | 1 513 | 2 108 | 2 097 | 2 277 | 31 459 | 41 963 | 1 501 | 845 |
|  | 1% | 1% | 1% | 1% | 9% | 10% | 1% | 1% |
