# Supplementary material for: Association between internal migration and epidemic dynamics: an analysis of cause-specific mortality in Kenya and South Africa using health and demographic surveillance data
Source: BMC Public Health. 2018 Jul 27;18:918. doi: 10.1186/s12889-018-5851-5 (PMC6062880; doi:10.1186/s12889-018-5851-5)
Supplement: Supplementary file 2 — Number and percentage of AIDS/TB and NCD deaths by HDSS site and sex. (DOCX 27 kb) [file 12889_2018_5851_MOESM2_ESM.docx]

**Table S2:** **Number and percentage of AIDS/TB and NCD deaths by HDSS site and sex**

|  | **Agincourt HDSS** | | **AHRI HDSS** | | **Kisumu HDSS** | | **Nairobi HDSS** | |
| --- | --- | --- | --- | --- | --- | --- | --- | --- |
|  | Male | Female | Male | Female | Male | Female | Male | Female |
| **Non-migrants** |  |  |  |  |  |  |  |  |
| Person Years | 93 551 | 125 691 | 109 304 | 149 530 | 259 193 | 304 621 | 83 473 | 54 691 |
| AIDS/TB | 274 | 556 | 1034 | 1306 | 1480 | 1785 | 292 | 228 |
| NCDs | 123 | 239 | 267 | 300 | 748 | 619 | 107 | 86 |
| Other | 257 | 198 | 432 | 288 | 1146 | 1060 | 297 | 127 |
| **In-migrants** |  |  |  |  |  |  |  |  |
| Person Years | 59 399 | 109 805 | 37 458 | 49 812 | 63 390 | 88 512 | 89 428 | 64 961 |
| AIDS/TB | 370 | 637 | 484 | 582 | 442 | 585 | 143 | 190 |
| NCDs | 117 | 162 | 77 | 100 | 171 | 120 | 39 | 48 |
| Other | 233 | 218 | 182 | 89 | 326 | 363 | 310 | 135 |
| **Return migrants** | |  |  |  |  |  |  |  |
| Person Years | 13 965 | 11 320 | 20 844 | 25 381 | 32 089 | 32 131 | 19 932 | 14 684 |
| AIDS/TB | 524 | 275 | 217 | 265 | 213 | 200 | 71 | 57 |
| NCDs | 197 | 75 | 31 | 27 | 71 | 39 | 19 | 15 |
| Other | 375 | 107 | 124 | 57 | 159 | 96 | 110 | 40 |
| **Total residents** |  |  |  |  |  |  |  |  |
| Person Years | 166 915 | 246 816 | 167 606 | 224 723 | 354 672 | 425 264 | 192 833 | 134 336 |
| AIDS/TB | 1168 | 1468 | 1735 | 2153 | 2135 | 2570 | 506 | 475 |
|  | 47% | 60% | 61% | 71% | 45% | 53% | 36% | 51% |
| NCDs | 437 | 476 | 375 | 427 | 990 | 778 | 165 | 149 |
|  | 18% | 19% | 13% | 14% | 21% | 16% | 12% | 16% |
| Other | 865 | 523 | 738 | 434 | 1631 | 1519 | 717 | 302 |
|  | 35% | 21% | 26% | 14% | 34% | 31% | 52% | 33% |
| **Total Deaths** | 2470 | 2467 | 2848 | 3014 | 4756 | 4867 | 1388 | 926 |
