# Supplementary material for: Association between internal migration and epidemic dynamics: an analysis of cause-specific mortality in Kenya and South Africa using health and demographic surveillance data
Source: BMC Public Health. 2018 Jul 27;18:918. doi: 10.1186/s12889-018-5851-5 (PMC6062880; doi:10.1186/s12889-018-5851-5)
Supplement: Supplementary file 3 — Agincourt HDSS Competing Risk Models. (DOCX 31 kb) [file 12889_2018_5851_MOESM3_ESM.docx]

**Table S3: Agincourt HDSS Competing Risk Models^a^**

|  | **AIDS/TB Males** | **AIDS/TB Females** | **NCDs Males** | **NCDs Females** |
| --- | --- | --- | --- | --- |
| **Duration since in-migration** |  |  |  |  |
| 2-5y in-migrant | 0.80* | 0.77*** | 0,98 | 0,78 |
|  | (0.62 - 1.01) | (0.64 - 0.93) | (0.63 - 1.51) | (0.52 - 1.15) |
| 5-9y in-migrant | 0.64*** | 0.72*** | 0,81 | 0,86 |
|  | (0.50 - 0.83) | (0.59 - 0.87) | (0.51 - 1.28) | (0.59 - 1.26) |
| **Duration since return migration** |  |  |  |  |
| 2-5y return migrant | 0.35*** | 0.39*** | 0.42*** | 0.37*** |
|  | (0.28 - 0.44) | (0.29 - 0.52) | (0.29 - 0.61) | (0.21 - 0.67) |
| 5-9y return migrant | 0.27*** | 0.23*** | 0.46*** | 0.20*** |
|  | (0.19 - 0.37) | (0.14 - 0.38) | (0.29 - 0.71) | (0.08 - 0.50) |
| **Return Migrant Exposure <36months** |  |  |  |  |
| 36+ months away | 1.49*** | 1.52*** | 1,04 | 1,35 |
|  | (1.19 - 1.86) | (1.18 - 1.94) | (0.74 - 1.45) | (0.81 - 2.24) |
| **Period** |  |  |  |  |
| 1998 – 2000 | 0.62** | 0.73* | 1,33 | 0,71 |
|  | (0.40 - 0.95) | (0.52 - 1.03) | (0.72 - 2.45) | (0.47 - 1.10) |
| 2001 – 2003 | 1.57** | 1.66*** | 1,25 | 1,07 |
|  | (1.07 - 2.32) | (1.23 - 2.24) | (0.64 - 2.45) | (0.72 - 1.58) |
| 2004 – 2006 | 1.86*** | 2.45*** | 2.36*** | 0,99 |
|  | (1.27 - 2.73) | (1.85 - 3.24) | (1.30 - 4.30) | (0.67 - 1.47) |
| 2007 – 2009 | 1,33 | 2.06*** | 1,62 | 1,04 |
|  | (0.88 - 2.00) | (1.55 - 2.74) | (0.86 - 3.06) | (0.71 - 1.53) |
| 2010 - 2012 (Ref) | 1 | 1 | 1 | 1 |
| **Migrant status 1998 - 2000** |  |  |  |  |
| In-migrant | 1,27 | 1,25 | 0.20*** | 0,96 |
|  | (0.80 - 2.02) | (0.86 - 1.83) | (0.07 - 0.57) | (0.52 - 1.77) |
| Return migrant | 5.05*** | 3.73*** | 3.56*** | 4.16*** |
|  | (3.19 - 7.99) | (1.70 - 8.17) | (1.98 - 6.38) | (1.64 - 10.52) |
| **Migrant status 2001 - 2003** |  |  |  |  |
| In-migrant | 1.51** | 1.37** | 1,36 | 1,03 |
|  | (1.06 - 2.16) | (1.04 - 1.80) | (0.69 - 2.69) | (0.61 - 1.72) |
| Return migrant | 4.02*** | 5.00*** | 3.92*** | 2,02 |
|  | (2.80 - 5.76) | (3.37 - 7.44) | (2.04 - 7.56) | (0.82 - 4.97) |
| **Migrant status 2004 - 2006** |  |  |  |  |
| In-migrant | 1.57** | 1.37** | 1,05 | 1,03 |
|  | (1.11 - 2.21) | (1.07 - 1.76) | (0.61 - 1.83) | (0.61 - 1.75) |
| Return migrant | 4.37*** | 5.14*** | 3.57*** | 3.40*** |
|  | (3.08 - 6.19) | (3.77 - 7.00) | (2.15 - 5.93) | (1.86 - 6.23) |
| **Migrant status 2007 - 2009** |  |  |  |  |
| In-migrant | 1.99*** | 1,13 | 1,11 | 1,13 |
|  | (1.39 - 2.85) | (0.87 - 1.47) | (0.59 - 2.08) | (0.69 - 1.84) |
| Return migrant | 6.24*** | 4.09*** | 4.40*** | 2.81*** |
|  | (4.28 - 9.08) | (2.88 - 5.80) | (2.48 - 7.79) | (1.40 - 5.63) |
| **Migrant status 2010 - 2012** |  |  |  |  |
| In-migrant | 1.47* | 1.46** | 2.39*** | 1,26 |
|  | (0.96 - 2.24) | (1.04 - 2.03) | (1.25 - 4.57) | (0.77 - 2.06) |
| Return migrant | 3.08*** | 4.59*** | 5.18*** | 2.87*** |
|  | (2.00 - 4.74) | (3.09 - 6.82) | (2.76 - 9.74) | (1.44 - 5.71) |
| **Education** |  |  |  |  |
| No Formal (Ref) | 1 | 1 | 1 | 1 |
| Some Primary | 1 | 0.87* | 0,95 | 1,07 |
|  | (0.84 - 1.19) | (0.74 - 1.02) | (0.71 - 1.26) | (0.82 - 1.40) |
| Some Secondary | 0.68*** | 0.77*** | 0,86 | 1,13 |
|  | (0.57 - 0.82) | (0.67 - 0.90) | (0.65 - 1.15) | (0.87 - 1.46) |
| Some Tertiary | 0.29*** | 0.29*** | 0.49*** | 0,81 |
|  | (0.21 - 0.41) | (0.21 - 0.39) | (0.29 - 0.82) | (0.52 - 1.26) |
| Unknown | 2.11*** | 2.81*** | 1.95** | 2.51*** |
|  | (1.47 - 3.03) | (2.00 - 3.95) | (1.04 - 3.67) | (1.27 - 4.95) |
| Observations | 310 399 | 441 421 | 310 399 | 441 421 |
| Wald Chi-square | 860,4 | 900,1 | 203,3 | 110,4 |
| Log Likelihood | -8522 | -12127 | -3233 | -3928 |
| Subjects | 39089 | 53114 | 39089 | 53114 |
| Failures | 1168 | 1468 | 437 | 476 |
| *** p<0.01, ** p<0.05, * p<0.1 |  |  |  |  |

**^a^** Example of how to interpret the table: to calculate the relative risk of a male return migrant (who has spent 6 years in the HDSS since his return, after being away for more than 4 years) dying from AIDS/TB during the period 2004 – 2006, with a non-migrant during the same period with the same level of education, the following formula would be applied: 4.37*0.27*1.49 = 1.76. i.e.: the risk of this migrant dying from AIDS/TB is 1.76 times more than a non-migrants of the same education level in the same period.
