# Supplementary material for: Association between internal migration and epidemic dynamics: an analysis of cause-specific mortality in Kenya and South Africa using health and demographic surveillance data
Source: BMC Public Health. 2018 Jul 27;18:918. doi: 10.1186/s12889-018-5851-5 (PMC6062880; doi:10.1186/s12889-018-5851-5)
Supplement: Supplementary file 4 — Africa Health Research Institute HDSS Competing Risk Models. (DOCX 30 kb) [file 12889_2018_5851_MOESM4_ESM.docx]

**Table S4: Africa Health Research Institute HDSS Competing Risk Models**

|  | **AIDS/TB Males** | **AIDS/TB Females** | **NCDs Males** | **NCDs Females** |
| --- | --- | --- | --- | --- |
| **Duration since in-migration** |  |  |  |  |
| 2-5y in-migrant | 0.81* | 1,1 | 1,21 | 1,55 |
|  | (0.66 - 1.01) | (0.91 - 1.35) | (0.69 - 2.12) | (0.88 - 2.76) |
| 5-9y in-migrant | 0,85 | 1,2 | 1,54 | 1.89** |
|  | (0.65 - 1.11) | (0.93 - 1.54) | (0.84 - 2.85) | (1.01 - 3.52) |
| **Duration since return migration** |  |  |  |  |
| 2-5y return migrant | 0,99 | 0,86 | 1,63 | 1,35 |
|  | (0.73 - 1.34) | (0.66 - 1.11) | (0.82 - 3.24) | (0.57 - 3.23) |
| 5-9y return migrant | 1,1 | 0.69* | 0,66 | 1,18 |
|  | (0.74 - 1.62) | (0.47 - 1.03) | (0.17 - 2.52) | (0.33 - 4.23) |
| **Return Migrant Exposure <36months** |  |  |  |  |
| 36+ months away | 1,26 | 1,14 | 0,63 | 1,01 |
|  | (0.92 - 1.75) | (0.85 - 1.53) | (0.23 - 1.71) | (0.38 - 2.67) |
| **Period** |  |  |  |  |
| 1998 – 2000 | -- | -- | -- | -- |
|  |  |  |  |  |
| 2001 – 2003 | 1.51*** | 2.13*** | 0,85 | 0,76 |
|  | (1.23 - 1.85) | (1.76 - 2.58) | (0.57 - 1.26) | (0.53 - 1.09) |
| 2004 – 2006 | 1.54*** | 2.24*** | 1.49** | 1.43** |
|  | (1.24 - 1.91) | (1.83 - 2.73) | (1.01 - 2.20) | (1.02 - 2.01) |
| 2007 – 2009 | 1,19 | 1.44*** | 1.49* | 1.39* |
|  | (0.94 - 1.50) | (1.16 - 1.79) | (1.00 - 2.24) | (0.99 - 1.97) |
| 2010 - 2012 (Ref) | 1 | 1 | 1 | 1 |
| **Migrant status 1998 - 2000** |  |  |  |  |
| In-migrant | -- | -- | -- | -- |
|  |  |  |  |  |
| Return migrant | -- | -- | -- | -- |
| **Migrant status 2001 - 2003** |  |  |  |  |
| In-migrant | 1,12 | 1,01 | 0.32*** | 0.44** |
|  | (0.90 - 1.40) | (0.82 - 1.23) | (0.14 - 0.76) | (0.20 - 0.96) |
| Return migrant | 1,47 | 1,36 | 0.00*** | 0.00*** |
|  | (0.68 - 3.19) | (0.68 - 2.72) | (0.00 - 0.00) | (0.00 - 0.00) |
| **Migrant status 2004 - 2006** |  |  |  |  |
| In-migrant | 1,07 | 1,11 | 0.49** | 0,79 |
|  | (0.83 - 1.36) | (0.90 - 1.38) | (0.26 - 0.93) | (0.43 - 1.44) |
| Return migrant | 1,13 | 1.58*** | 0,8 | 0,71 |
|  | (0.79 - 1.62) | (1.18 - 2.12) | (0.33 - 1.95) | (0.26 - 1.93) |
| **Migrant status 2007 - 2009** |  |  |  |  |
| In-migrant | 1.50*** | 1,2 | 0,74 | 0,74 |
|  | (1.15 - 1.95) | (0.92 - 1.56) | (0.42 - 1.30) | (0.39 - 1.40) |
| Return migrant | 1,12 | 1.86*** | 0.50* | 0,73 |
|  | (0.79 - 1.59) | (1.41 - 2.47) | (0.22 - 1.12) | (0.31 - 1.71) |
| **Migrant status 2010 - 2012** |  |  |  |  |
| In-migrant | 1.30* | 1,07 | 0.47** | 0,67 |
|  | (0.96 - 1.78) | (0.78 - 1.47) | (0.23 - 0.97) | (0.34 - 1.32) |
| Return migrant | 1,07 | 1.80*** | 0,67 | 0,6 |
|  | (0.76 - 1.51) | (1.30 - 2.49) | (0.31 - 1.47) | (0.20 - 1.81) |
| **Education** |  |  |  |  |
| No Formal (Ref) | 1 | 1 | 1 | 1 |
| Some Primary | 1.36*** | 0,95 | 0,94 | 1,11 |
|  | (1.12 - 1.66) | (0.80 - 1.14) | (0.66 - 1.34) | (0.80 - 1.55) |
| Some Secondary | 0,85 | 0.70*** | 0.65** | 0,88 |
|  | (0.69 - 1.04) | (0.58 - 0.84) | (0.45 - 0.96) | (0.61 - 1.26) |
| Some Tertiary | 0.29*** | 0.19*** | 0.41*** | 0.61** |
|  | (0.23 - 0.36) | (0.15 - 0.24) | (0.28 - 0.62) | (0.40 - 0.93) |
| Unknown | 4.21*** | 5.71*** | 3.52*** | 7.53*** |
|  | (3.36 - 5.26) | (4.62 - 7.06) | (2.32 - 5.35) | (4.97 - 11.40) |
| Observations | 124 861 | 163 608 | 124 861 | 163 608 |
| Wald Chi-square | 890,9 | 1553 | 2335 | 5462 |
| Log Likelihood | -13206 | -17465 | -2857 | -3470 |
| Subjects | 36755 | 43414 | 36755 | 43414 |
| Failures | 1735 | 2153 | 375 | 427 |
| *** p<0.01, ** p<0.05, * p<0.1 |  |  |  |  |
|  |  |  |  |  |
