# Supplementary material for: Association between internal migration and epidemic dynamics: an analysis of cause-specific mortality in Kenya and South Africa using health and demographic surveillance data
Source: BMC Public Health. 2018 Jul 27;18:918. doi: 10.1186/s12889-018-5851-5 (PMC6062880; doi:10.1186/s12889-018-5851-5)
Supplement: Supplementary file 5 — Kisumu HDSS Competing Risk Models. (DOCX 29 kb) [file 12889_2018_5851_MOESM5_ESM.docx]

**Table S5: Kisumu HDSS Competing Risk Models**

|  | **AIDS/TB Males** | **AIDS/TB Females** | **NCDs Males** | **NCDs Females** |
| --- | --- | --- | --- | --- |
| **Duration since in-migration** |  |  |  |  |
| 2-5y in-migrant | 0.78** | 0.84** | 1,26 | 0,86 |
|  | (0.63 - 0.96) | (0.70 - 1.00) | (0.91 - 1.75) | (0.58 - 1.26) |
| 5-9y in-migrant | 0,82 | 0.68*** | 0,92 | 0,87 |
|  | (0.60 - 1.11) | (0.51 - 0.90) | (0.54 - 1.57) | (0.50 - 1.51) |
| **Duration since return migration** |  |  |  |  |
| 2-5y return migrant | 1,1 | 0.76* | 1,33 | 0,66 |
|  | (0.82 - 1.49) | (0.54 - 1.05) | (0.79 - 2.25) | (0.33 - 1.31) |
| 5-9y return migrant | 1,05 | 0,65 | 0,95 | 0,6 |
|  | (0.66 - 1.67) | (0.36 - 1.18) | (0.37 - 2.40) | (0.17 - 2.10) |
| **Return Migrant Exposure <36months** |  |  |  |  |
| 36+ months away | 0,97 | 0,91 | 1,55 | 1,38 |
|  | (0.67 - 1.42) | (0.57 - 1.46) | (0.83 - 2.91) | (0.58 - 3.29) |
| **Period** |  |  |  |  |
| 1998 – 2000 | -- | -- | -- | -- |
|  |  |  |  |  |
| 2001 – 2003 | -- | -- | -- | -- |
|  |  |  |  |  |
| 2004 – 2006 | 1.99*** | 2.89*** | 1.68*** | 1.78*** |
|  | (1.74 - 2.27) | (2.55 - 3.28) | (1.39 - 2.04) | (1.45 - 2.20) |
| 2007 – 2009 | 1.54*** | 1.81*** | 1.60*** | 1.63*** |
|  | (1.35 - 1.75) | (1.59 - 2.06) | (1.34 - 1.92) | (1.33 - 1.99) |
| 2010 - 2012 (Ref) | 1 | 1 | 1 | 1 |
| **Migrant status 1998 - 2000** |  |  |  |  |
| In-migrant | -- | -- | -- | -- |
| Return migrant | -- | -- | -- | -- |
| **Migrant status 2001 - 2003** |  |  |  |  |
| In-migrant | -- | -- | -- | -- |
|  |  |  |  |  |
| Return migrant | -- | -- | -- | -- |
| **Migrant status 2004 - 2006** |  |  |  |  |
| In-migrant | 1.61*** | 1.72*** | 1,18 | 1.59** |
|  | (1.32 - 1.95) | (1.44 - 2.05) | (0.85 - 1.65) | (1.05 - 2.41) |
| Return migrant | 1,15 | 2.06*** | 1,39 | 1,13 |
|  | (0.74 - 1.80) | (1.50 - 2.83) | (0.73 - 2.65) | (0.42 - 3.08) |
| **Migrant status 2007 - 2009** |  |  |  |  |
| In-migrant | 1.40*** | 1.89*** | 0,86 | 1.63*** |
|  | (1.13 - 1.72) | (1.58 - 2.26) | (0.62 - 1.20) | (1.13 - 2.36) |
| Return migrant | 1.31* | 1.83*** | 0,78 | 1.70* |
|  | (0.97 - 1.76) | (1.39 - 2.43) | (0.47 - 1.30) | (0.96 - 3.00) |
| **Migrant status 2010 - 2012** |  |  |  |  |
| In-migrant | 1.28** | 1.69*** | 0,78 | 1,16 |
|  | (1.00 - 1.62) | (1.36 - 2.09) | (0.53 - 1.17) | (0.76 - 1.78) |
| Return migrant | 1.37** | 1.69*** | 0,77 | 1,44 |
|  | (1.01 - 1.85) | (1.20 - 2.38) | (0.44 - 1.37) | (0.73 - 2.85) |
| **Education** |  |  |  |  |
| No Formal (Ref) | 1 | 1 | 1 | 1 |
| Some Primary | 0,9 | 0.78*** | 0.43*** | 0.70*** |
|  | (0.67 - 1.20) | (0.67 - 0.90) | (0.32 - 0.58) | (0.57 - 0.86) |
| Some Secondary | 0.56*** | 0.45*** | 0.36*** | 0.46*** |
|  | (0.41 - 0.77) | (0.37 - 0.55) | (0.26 - 0.50) | (0.33 - 0.64) |
| Some Tertiary | 0.30*** | 0.15*** | 0.24*** | 0.38*** |
|  | (0.20 - 0.44) | (0.08 - 0.27) | (0.16 - 0.38) | (0.19 - 0.79) |
| Unknown | 0,95 | 0,9 | 0.48*** | 0.69*** |
|  | (0.70 - 1.30) | (0.75 - 1.06) | (0.35 - 0.67) | (0.53 - 0.91) |
| Observations | 275 548 | 331 145 | 275 548 | 331 145 |
| Wald Chi-square | 334,5 | 631,9 | 125,4 | 98,22 |
| Log Likelihood | -18359 | -22984 | -8550 | -6951 |
| Subjects | 93177 | 115174 | 93177 | 115174 |
| Failures | 2135 | 2570 | 990 | 778 |
| *** p<0.01, ** p<0.05, * p<0.1 |  |  |  |  |
|  |  |  |  |  |
