# Supplementary material for: Association between internal migration and epidemic dynamics: an analysis of cause-specific mortality in Kenya and South Africa using health and demographic surveillance data
Source: BMC Public Health. 2018 Jul 27;18:918. doi: 10.1186/s12889-018-5851-5 (PMC6062880; doi:10.1186/s12889-018-5851-5)
Supplement: Supplementary file 6 — Nairobi HDSS Competing Risk Models. (DOCX 30 kb) [file 12889_2018_5851_MOESM6_ESM.docx]

**Table S6: Nairobi HDSS Competing Risk Models**

|  | **AIDS/TB Males** | **AIDS/TB Females** | **NCDs Males** | **NCDs Females** |
| --- | --- | --- | --- | --- |
| **Duration since in-migration** |  |  |  |  |
| 2-5y in-migrant | 1,15 | 0,8 | 1,21 | 0.39** |
|  | (0.80 - 1.65) | (0.59 - 1.09) | (0.60 - 2.46) | (0.19 - 0.81) |
| 5-9y in-migrant | 0,94 | 0.64* | 0,41 | 0,5 |
|  | (0.56 - 1.56) | (0.39 - 1.03) | (0.10 - 1.65) | (0.19 - 1.30) |
| **Duration since return migration** |  |  |  |  |
| 2-5y return migrant | 0,87 | 1,04 | 0,8 | 0,44 |
|  | (0.52 - 1.44) | (0.52 - 2.06) | (0.30 - 2.14) | (0.13 - 1.45) |
| 5-9y return migrant | 0,82 | 1,54 | 1,19 | 0,61 |
|  | (0.37 - 1.84) | (0.68 - 3.48) | (0.38 - 3.72) | (0.12 - 3.07) |
| **Return Migrant Exposure <36months** |  |  |  |  |
| 36+ months away | 1,59 | 0,97 | 1,61 | 2,12 |
|  | (0.82 - 3.07) | (0.35 - 2.67) | (0.52 - 4.95) | (0.66 - 6.82) |
| **Period** |  |  |  |  |
| 1998 – 2000 | -- | -- | -- | -- |
|  |  |  |  |  |
| 2001 – 2003 | -- | -- | -- | -- |
|  |  |  |  |  |
| 2004 – 2006 | 1.93*** | 2.26*** | 0,86 | 1,07 |
|  | (1.42 - 2.62) | (1.57 - 3.24) | (0.53 - 1.39) | (0.63 - 1.84) |
| 2007 – 2009 | 1,12 | 1.49* | 1,15 | 1,19 |
|  | (0.79 - 1.58) | (1.00 - 2.22) | (0.72 - 1.85) | (0.69 - 2.05) |
| 2010 - 2012 (Ref) | 1 | 1 | 1 | 1 |
| **Migrant status 1998 - 2000** |  |  |  |  |
| In-migrant | -- | -- | -- | -- |
|  |  |  |  |  |
| Return migrant | -- | -- | -- | -- |
| **Migrant status 2001 - 2003** |  |  |  |  |
| In-migrant | -- | -- | -- | -- |
|  |  |  |  |  |
| Return migrant | -- | -- | -- | -- |
|  |  |  |  |  |
| **Migrant status 2004 - 2006** |  |  |  |  |
| In-migrant | 0.61** | 1,16 | 0,69 | 1,64 |
|  | (0.41 - 0.92) | (0.81 - 1.66) | (0.34 - 1.40) | (0.86 - 3.12) |
| Return migrant | 1,13 | 0,81 | 0.00*** | 1,08 |
|  | (0.60 - 2.12) | (0.40 - 1.66) | (0.00 - 0.00) | (0.26 - 4.52) |
| **Migrant status 2007 - 2009** |  |  |  |  |
| In-migrant | 0,83 | 1,1 | 0.34*** | 0,69 |
|  | (0.53 - 1.31) | (0.75 - 1.63) | (0.16 - 0.72) | (0.32 - 1.48) |
| Return migrant | 1,46 | 0,87 | 0,48 | 1,25 |
|  | (0.87 - 2.45) | (0.45 - 1.69) | (0.16 - 1.43) | (0.51 - 3.07) |
| **Migrant status 2010 - 2012** |  |  |  |  |
| In-migrant | 0,81 | 1.86*** | 0,56 | 1,4 |
|  | (0.53 - 1.24) | (1.20 - 2.90) | (0.25 - 1.28) | (0.69 - 2.85) |
| Return migrant | 1.71* | 1,61 | 1,21 | 1,12 |
|  | (0.94 - 3.11) | (0.75 - 3.45) | (0.50 - 2.88) | (0.36 - 3.47) |
| **Education** |  |  |  |  |
| No Formal (Ref) | 1 | 1 | 1 | 1 |
| Some Primary | 1,12 | 0,85 | 1,71 | 0,68 |
|  | (0.72 - 1.75) | (0.62 - 1.16) | (0.69 - 4.24) | (0.42 - 1.08) |
| Some Secondary | 0,74 | 0.41*** | 0,98 | 0.51** |
|  | (0.46 - 1.17) | (0.28 - 0.60) | (0.38 - 2.50) | (0.29 - 0.91) |
| Some Tertiary | 0,52 | 0.00*** | 0,73 | 0.00*** |
|  | (0.15 - 1.76) | (0.00 - 0.00) | (0.08 - 6.29) | (0.00 - 0.00) |
| Unknown | 3.31*** | 1,56 | 1,14 | 1,51 |
|  | (1.68 - 6.49) | (0.70 - 3.49) | (0.13 - 9.68) | (0.35 - 6.57) |
| Observations | 362 683 | 262 038 | 362 683 | 262 038 |
| Wald Chi-square | 97,13 | 22225 | 23984 | 8488 |
| Log Likelihood | -4122 | -3772 | -1337 | -1130 |
| Subjects | 67145 | 49617 | 67145 | 49617 |
| Failures | 506 | 475 | 165 | 149 |
| *** p<0.01, ** p<0.05, * p<0.1 |  |  |  |  |
|  |  |  |  |  |
